# Editorial: Inflammasome, purinergic signaling, and immunometabolism in oral health and disease

**DOI:** 10.3389/froh.2023.1236507

**Published:** 2023-06-16

**Authors:** Ana Carolina Morandini, Luiz Eduardo Baggio Savio

**Affiliations:** ^1^ Department of Oral Biology and Diagnostic Sciences, Dental College of Georgia at Augusta University, Augusta, GA, United States; ^2^ Department of Periodontics, Dental College of Georgia at Augusta University, Augusta, GA, United States; ^3^ Instituto de Biofísica Carlos Chagas Filho, Universidade Federal do Rio de Janeiro (UFRJ), Rio de Janeiro, Brazil

**Keywords:** inflammasome, purinergic signaling, immunometabolism, periodontitis, oral health, NLRP3 inflammasome, adenosine triphosphate

**Editorial on the Research Topic** Inflammasome, purinergic signaling, and immunometabolism in oral health and disease

Emerging evidence suggests a complex interplay between inflammasome activation, purinergic signaling, and immunometabolism, highlighting their interconnectedness in immune regulation. Purinergic signaling can regulate inflammasome activation by modulating Adenosine triphosphate (ATP) release or its metabolism. ATP released during inflammation can activate the NLRP3 inflammasome, promoting the maturation and secretion of pro-inflammatory cytokines (1). The NLRP3 inflammasome is a multi-protein complex, which is activated upon bacterial infections or cellular damage. Upon activation, pro-caspase1 is cleaved to active caspase1, which then proceeds to cleave the cytokine precursors pro-IL-1β and pro-IL-18 into their mature secreted forms (2). Additionally, inflammasome activation can influence purinergic signaling by affecting ATP release or enzymatic activities involved in ATP breakdown, altering the balance between pro-inflammatory ATP signaling and immunosuppressive adenosine signaling (3).

NLRP3 inflammasome is essential for defending against bacterial infections and misregulated NLRP3 inflammasome has been implicated in metabolic inflammatory disorders including type 2 diabetes, atherosclerosis, chronic kidney diseases (4), and respiratory disorders as reviewed by Leszczyńska et al. The authors elegantly discuss the contribution of NLRP3 inflammasome to the development of allergic rhinitis, allergic asthma, and chronic obstructive pulmonary disease. Huang et al. discussed the metals, which are widely used in dental implant manufacturing and dental crowns for oral rehabilitation in regards to their involvement in the activation of NLRP3 inflammasome. In this context, the cathepsins are a family of proteases that have been implicated in NLRP3 inflammasome activation following their activation with ATP, monosodium urate, silica crystals, or bacterial components, among others (5). In this regard, Jiang et al. explored the recent evidence in the pathogenic mechanisms between cathepsins and the most common oral diseases such as dental caries, periodontitis, oral cancer, and periapical lesions.

Metabolic reprogramming is a well-developed concept pertaining to changes in cellular bioenergetics (6) which has been embraced by the immunology field giving rise to immunometabolism (7) as one of the phenomena influencing intracellular pathways such as inflammasome activation and purinergic signaling. Metabolic intermediates can directly affect inflammasome assembly and cytokine production, while changes in energy metabolism can influence ATP release and cell death. In particular, ferroptosis, a type of iron-dependent autophagy-related cell death (8, 9) has been described by Xie et al. underlying the pathological and functional mechanisms underlying ferroptosis in irreversible pulpitis. Furthermore, Liu et al. explored a recently discovered cell death pathway initiated by copper ion clusters named cuproptosis using a wide array of cutting-edge bioinformatics approaches to evaluate existing human gingival tissue gene expression datasets, thus providing an innovative possible connection between cuproptosis and periodontitis. Finally, Hou et al. provide a comprehensive review on the relationship between high levels of uric acid and periodontitis. The authors discuss the pathogenic mechanisms driving periodontitis and the systemic impact of hyperuricemia, and summarize the link between the two disease mechanisms, wrapping up this special research topic.

In summary, this Special Issue offers a great overview of how inflammasome, purinergic signaling, and immunometabolism affect oral health and disease. The points discussed in this issue open new avenues for future research on these signaling pathways to better understand the physiological and pathological mechanisms underlying oral disease.
